# Climate for evidence-informed health systems: A print media analysis in 44 low- and middle-income countries that host knowledge-translation platforms

**DOI:** 10.1186/1478-4505-9-7

**Published:** 2011-02-08

**Authors:** Andrew Cheung, John N Lavis, Ali Hamandi, Fadi El-Jardali, Jonathan Sachs, Nelson Sewankambo

**Affiliations:** 1 Faculty of Medicine, University of Ottawa, 451 Smyth Rd., Ottawa, Ontario, K1H 8M5, Canada; 2 McMaster Health Forum, MML-417, 1280 Main St. West, Hamilton, Ontario, L8S 4L6, Canada; 3 Centre for Health Economics and Policy Analysis, McMaster University, CRL-209, 1280 Main St. West, Hamilton, Ontario, L8S 4K1, Canada; 4 Department of Clinical Epidemiology and Biostatistics, McMaster University, CRL-209, 1280 Main St. West, Hamilton, Ontario, L8S 4K1, Canada; 5 Department of Political Science, McMaster University, McMaster University, CRL-209, 1280 Main St. West, Hamilton, Ontario, L8S 4K1, Canada; 6 Harvard School of Public Health, 677 Huntington Ave., Boston, MA, 02115, USA; 7 Department of Health Management and Policy, American University of Beirut, Room 107C, PO Box 11-0236, Riad El Solh, Beirut, 1107 2020, Lebanon; 8 Canadian Health Services Research Foundation, 1565 Carling Avenue, Suite 700, Ottawa, Ontario, K1Z 8R1, Canada; 9 College of Health Sciences, Makerere University, PO Box 7072, Kampala, Uganda; 10 Centre for the Development of Best Practices in Health, Yaounde Central Hospital, PO Box 5604, Yaounde, Cameroon; 11 Departamento Medicina Familiar, Pontificia Universidad Católica de Chile, Lira 44, Edificio Decanato, Primer Piso, Santiago, Chile; 12 Institute for Health Systems Research, Ministry of Health, Jalan Rumah Sakit Bangsar, 59000, Kuala Lumpur, Malaysia

## Abstract

**Background:**

We conducted a print media analysis in 44 countries in Africa, the Americas, Asia, and the Eastern Mediterranean in order to understand one dimension of the climate for evidence-informed health systems and to provide a baseline for an evaluation of knowledge-translation platforms. Our focus was whether and how policymakers, stakeholders, and researchers talk in the media about three topics: policy priorities in the health sector, health research evidence, and policy dialogues regarding health issues.

**Methods:**

We developed a search strategy consisting of three progressively more delimited phases. For each jurisdiction, we searched *Major World Publications *in LexisNexis Academic *News *for articles published in 2007, selected relevant articles using one set of general criteria and three sets of concept-specific criteria, and coded the selected articles to identify common themes. Second raters took part in the analysis of Lebanon and Malaysia to assess inter-rater reliability for article selection and coding.

**Results:**

We identified approximately 5.5 and 5 times more articles describing health research evidence compared to the number of articles describing policy priorities and policy dialogues, respectively. Few articles describing health research evidence discussed systematic reviews (2%) or health systems research (2%), and few of the policy dialogue articles discussed researcher involvement (9%). News coverage of these concepts was highly concentrated in several countries like China and Uganda, while few articles were found for many other jurisdictions. Kappa scores were acceptable and consistently greater than 0.60.

**Conclusions:**

In many countries the print media, at least as captured in a global database, are largely silent about three topics central to evidence-informed health systems. These findings suggest the need for proactive-media engagement strategies.

## Introduction

Evidence-informed health systems refer to the systematic and transparent use of research evidence to strengthen health systems[1]. Policymakers need research evidence to inform decisions about what problems to focus on, what programs and services to offer, what governance, financial and delivery arrangements best support the provision of cost-effective programs and services, and what implementation strategies can get these programs and services to those who most need them[1].

A print media analysis can provide helpful insights to policymakers, stakeholders, researchers and journalists about one dimenstion of the climate for evidence-informed health systems[2]. Print media that cover policy priorities in the health sector are providing signals to researchers about topics for study[3]. Print media coverage can bring health research evidence to the attention of policymakers and stakeholders and convey to researchers that their work is valued[4]. Coverage of policy dialogues, which provide a forum in which policymakers, stakeholders and researchers learn from one another, can do the same[5]. Most of the insights that can be drawn about the climate for evidence-informed health systems are about the media's role as informers and agenda-setters, rather than as framers (i.e., agents that determine how issues are presented) or persuaders (i.e., agents that persuade the public regarding issues)[6]. Increases in media coverage over time may suggest that media-engagement strategies are working. On the other hand, a limited volume of print media coverage, or declines in coverage, may suggest the need for a proactive effort to engage the media.

**Figure 1:**
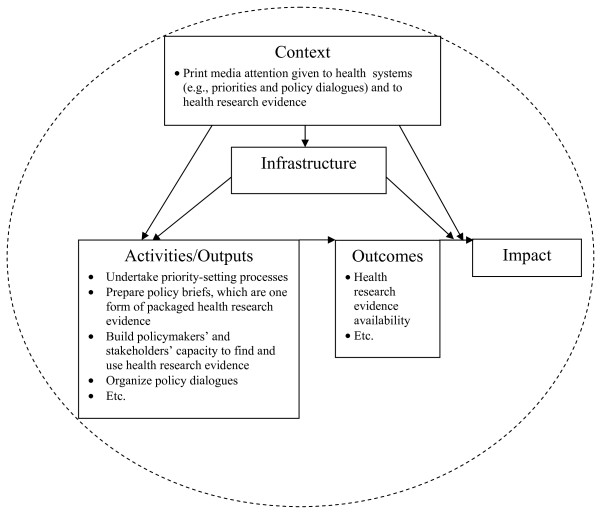
**Relationships between the focus of the print media analysis and the KT platform monitoring and evaluation framework**.

A print media analysis can also contribute to the monitoring and evaluation of the knowledge-translation (KT) platforms (i.e., entities devoted to fostering the use of research evidence in policymaking) called for in the 2004 Mexico Statement on Health Research, the 2005 World Health Assembly resolution, and the 2008 Bamako Call to Action on Research for Health[7-9]. For example, print media analysis is part of the monitoring and evaluation framework for the WHO-sponsored Evidence-Informed Policy Networks (EVIPNet) (Figure 1)[10]. Print media coverage can inform an understanding of the context in which KT platforms are operating, and this context may influence whether their activities and outputs translate into outcomes and impacts. Print media coverage can also provide an independent assessment of the KT platforms' activities (namely priority-setting processes and policy dialogues) and outputs (namely policy briefs, which are one form of packaged health research evidence),[11] as well as outcomes (namely that health research evidence about high-priority policy issues is more readily available).

In order to understand one dimension of the climate for evidence-informed health systems and to provide a baseline for an evaluation of KT platforms, we carried out a print media analysis in the 44 countries in Africa, the Americas, Asia, and the Eastern Mediterranean that host (or have signalled their intent to host) a local EVIPNet or similar type of KT platform[12,13]. In total we studied 47 jurisdictions because we included both China as a whole and the three Chinese provinces that host a local EVIPNet. The focus of our media representation study was whether and how policymakers, stakeholders, and researchers talk in the media about three topics: policy priorities in the health sector, health research evidence, and policy dialogues regarding health issues. For each topic we analyzed media messages for particular themes and constructs[14].

## Methods

We used LexisNexis Academic -- the world's largest online collection of news services - to conduct the print media analysis. It includes a comprehensive search function to identify specific subsets of articles. Despite its gaps in coverage, LexisNexis Academic has been shown to be the only viable option for conducting a cross-national print media analysis of this type, and there would have been many challenges associated with undertaking supplementary analyses of websites of local newspapers (e.g., they lack or have limited online search functions)[15]. Numerous studies have previously used LexisNexis Academic to examine media coverage of health issues, including subjects as diverse as discoveries of genetic links to breast and prostate cancer and citizens' views of long-term care[16,17]. We restricted our searches of LexisNexis Academic *News *to articles that had been published in the year 2007 in *Major World Publications*, which contains English-language sources. One author led the analysis of Africa, the Americas, and Asia, while another author led the effort in the Eastern Mediterranean. We categorized jurisdictions based on their geographical region rather than their WHO region.

### Developing a search strategy

We developed a three-phase search strategy for this analysis, which consisted of three sets of progressively more delimited search algorithms (Additional file 1). We developed the first set of searches by identifying search terms relevant to each of policy priorities, health research evidence, and policy dialogues, and combining these terms with the keywords *Health *and the name of the country or province (hereafter *Jurisdiction*). To assess this set of searches, we first identified a "gold standard" of relevant articles in Ethiopia and Cameroon (i.e., countries with English or both English and French among the languages spoken) by conducting a broad search for any articles containing the keyword *Health *and the name of the jurisdiction, and selecting articles that dealt with the three general concepts. We then examined how well our searches captured these "gold standard" articles, while also considering whether they returned a manageable number of results. We developed the second and third sets of gradually more restricted searches from our first set by searching for specific indexed terms, as opposed to looking for the terms anywhere in the articles. We employed the second and third sets of searches if the first and second sets, respectively, yielded more than 1500 results.

Since conducting this analysis, the user interface and search options in LexisNexis Academic *News *have changed. We have found that using the restrictions "everywhere" and "subject terms" in place of "anywhere in document" and "in indexing - any reference", respectively, yield highly comparable (but not identical) results to the search options used in this paper.

### Selecting relevant articles

In order to select articles that were relevant to our study, we used four sets of inclusion/exclusion criteria: one set of general criteria, and three sets of concept-specific criteria. We first applied the general criteria, which specified that an article was included if it focused on the jurisdiction being evaluated, and excluded if it was a duplicate. Afterwards, we applied the appropriate concept-specific criteria. We included articles that were found using the policy priorities algorithms if: 1) they mentioned a specific issue in the health sector as a government priority; and 2) the articulation of the priority was clearly attributable to the government, even if other groups had made this attribution. If articles only generally discussed issues without stating that they were government priorities, we excluded them. We included articles about health research evidence if they mentioned specific research studies addressing issues in the health sector, and we excluded them if they only cited statistics from international, governmental, or non-governmental organizations (i.e., they described data, not research per se). For articles retrieved using the policy dialogue searches, we included them if they described meetings that: 1) focused on health sector issues; and 2) brought together government officials with researchers or stakeholders or both. Articles that described conferences only attended by scientists or meetings only attended by stakeholders or government officials were excluded.

### Coding articles

We developed a coding form for each of policy priorities, health research evidence, and policy dialogues, and we coded the included articles to identify common themes[18]. For all included articles, we identified general characteristics, such as the title of the story, the publication date, and the source. For articles describing policy priorities in the health sector, we determined whether there were explicit mentions of a priority or priority setting process, declarations of specific targets related to the priority, or descriptions of who attributed the priority to the government. For articles addressing health research evidence, we examined the topics being studied (e.g., infectious diseases, chronic diseases, reproductive health), where the data were gathered, where researchers were based, the types of studies that were conducted (e.g., systematic reviews, randomized control trials), and whether or not the studies were peer-reviewed. In articles describing policy dialogues, we attempted to identify the location of the dialogue, the participants involved, the purpose of the dialogue, the party that initiated the dialogue, and any policy recommendations or agreements about actions resulting from the dialogues.

### Assessing inter-rater reliability

A single reviewer analyzed the articles retrieved for all 47 jurisdictions. Second reviewers independently applied the search strategy, inclusion/exclusion criteria, and coding protocol for the articles retrieved for Lebanon and Malaysia. Any disagreements that arose after applying the inclusion/exclusion criteria were resolved by consensus, and the included articles were coded. We calculated kappa scores to determine the level of inter-rater reliability for both the article selection and article coding phases, and sought to achieve at least substantial agreement (i.e., kappa exceeding 0.60).

## Results

Of the 32,938 articles initially retrieved by our search strategy, we identified 264 articles describing policy priorities in the health sector, 1468 describing health research evidence, and 290 articles describing policy dialogues that address health issues (Additional file 2). That is, we identified approximately 5.5 and 5 times more articles describing health research evidence compared to articles describing policy priorities and policy dialogues, respectively.

### Focus of articles

Of the 264 articles mentioning policy priorities, the majority of these priorities were attributed to the government by government officials themselves (n = 208, 79%), while stakeholders (n = 27, 10%) and researchers (n = 6, 2%) made such attributions less frequently (Table 1). Specific targets related to policy priorities were mentioned in 50 articles (19%). Africa and Asia accounted for the greatest number of articles describing policy priorities in the health sector (125 and 89, respectively), followed by the Eastern Mediterranean (n = 44) and the Americas (n = 6). For 30 of the 47 jurisdictions, we identified three or fewer articles describing health-related policy priorities.

**Table 1:** Findings from coding articles

Category	Topic	Africa	Americas	Asia	Eastern Mediterranean	Total
**Policy priorities**	Groups attributing priorities to the government					
	Government officials	98	5	77	28	**208**
	Researchers	2	0	3	1	**6**
	Stakeholders	15	1	6	5	**27**
						
	Articles mentioning specific targets	27	0	12	11	**50**
						
	**All articles describing specific policy priorities in the health sector**	**125**	**6**	**89**	**44**	**264**

**Health research evidence**	**Topics of research studies**||					
	Articles describing infectious disease research	275	121	132	55	**583**
	HIV/AIDS	188	30	26	14	**258**
	Malaria	49	5	2	1	**57**
	Tuberculosis	14	7	8	5	**34**
	Avian influenza	1	0	23	0	**24**
	Hepatitis (A, B, C)	2	4	11	1	**18**
	Schistosomiasis	2	4	4	0	**10**
	Chagas disease	0	7	0	0	**7**
	Dengue fever	0	2	2	0	**4**
	Other	34	67	70	35	**206**
	Articles describing chronic disease research	11	103	218	60	**392**
	Cancer	5	29	113	18	**165**
	Cardiovascular diseases	1	17	37	11	**66**
	Diabetes	2	17	16	6	**41**
	Obesity	0	13	18	1	**32**
	Neurodegenerative disorders	0	6	13	0	**19**
	Other	6	28	41	28	**103**
	Other research topics:					
	Reproductive health	61	48	38	36	**183**
	Mental health¶	14	45	28	9	**96**
	Nutrition	15	26	35	16	**92**
	Sexual health¶	10	6	18	13	**47**
	Complementary and alternative medicine¶	3	3	24	0	**30**
	Health systems¶	10	3	11	3	**27**
	Other	55	99	211	59	**424**
	**All articles specifying the topics of their research studies**	**389**	**356**	**523**	**200**	**1468**
						
	**Location of researchers described in articles**					
	Within jurisdiction	144	279	380	137	**940**
	Outside jurisdiction	142	75	117	46	**380**
	**All articles specifying the location of researchers**	**259**	**342**	**466**	**174**	**1241**
						
	**Types of studies**					
	Systematic reviews	3	11	11	6	**31**
	Randomized control trials	36	32	16	31	**115**
	Observational studies	30	66	67	22	**185**
	Studies in basic science	3	60	148	15	**226**
	Other	5	11	7	0	**23**
	**All articles specifying the types of studies**	**77**	**175**	**243**	**74**	**569**

**Policy dialogues**	Articles mentioning the participation of the following groups in policy dialogues					
	Government officials	154	8	101	24	**287**
	Researchers	19	0	7	1	**27**
	Stakeholders	151	8	100	24	**283**
						
	Articles describing policy dialogues that resulted in policy recommendations or agreements to action	48	4	32	10	**94**
						
	**All articles describing policy dialogues that address health issues**	**154**	**8**	**104**	**24**	**290**

||Articles could be coded to multiple topics

¶These topics were coded thematically

Of the 1468 articles we found describing health research evidence, African jurisdictions accounted for 389 articles, jurisdictions in the Americas accounted for 356 articles, Asian jurisdictions accounted for 523 articles, and Eastern Mediterranean jurisdictions accounted for 200 articles. Our analysis of these articles indicate that much of the research being covered addressed infectious diseases (n = 583, 40%) and chronic diseases (n = 392, 27%). Only 27 articles mentioned health systems research (2%). There appear to be some regional differences, however, as African jurisdictions accounted for far more articles describing infectious diseases research (n = 275, 71%), while a large portion of the articles relating to Asian jurisdictions featured research on chronic diseases (n = 218, 42%). Of the 1241 articles mentioning the location of researchers, 940 indicated that local researchers were involved in the research (76%), while 380 mentioned the participation of researchers from outside the jurisdiction (31%). Many of the articles mentioned the type of study being conducted (n = 569, 39%), and many of these articles described studies in the basic sciences (n = 226, 40%). Numerous other articles described observational studies (n = 185, 33%) and randomized control trials (n = 115, 20%), while only 31 discussed systematic reviews (5%). For 10 of the 47 jurisdictions, we identified three or fewer articles describing health research evidence.

Of the 290 articles describing policy dialogues addressing issues in the health sector, African and Asian jurisdictions accounted for the largest number of articles (154 and 104, respectively), and fewer articles were identified for the Eastern Mediterranean (n = 24) and the Americas (n = 8). Almost all of the policy dialogues described in the print media involved government officials (n = 287, 99%) and stakeholders (n = 283, 98%), while researcher involvement was noted in only 27 of the articles identified (9%). Some form of policy recommendation or agreement about action was described in 94 articles (32%). For 32 of the 47 jurisdictions, we identified three or fewer articles describing policy dialogues.

### Inter-rater reliability assessments

For the article-selection process, we achieved kappa scores that exceeded the threshold of 0.60 in our assessments of Lebanon, and in our assessments of health research evidence and policy dialogues for Malaysia. For our selection of articles describing policy priorities in Malaysia, we initially achieved a kappa score of only 0.47. However, upon re-assessing one-fifth of the articles, we achieved a kappa score of 0.84, indicating excellent agreement. In our assessment of inter-rater reliability for the coding protocol, we again achieved kappa scores that exceeded the substantial threshold of 0.60 for all coding categories.

## Discussions

### Principal findings

Our print media analysis in the year 2007 suggests that the climate for evidence-informed health systems can likely be improved in these 44 low- and middle-income countries (or 47 jurisdictions), at least in those countries whose media are well covered in the *Major World Publications *component of LexisNexis Academic *News*. However, the analysis provides a baseline for the within-country dimension of the monitoring and evaluation of the KT platforms located in these countries (with cross-country comparisons limited by likely differences in database coverage). We identified a larger number of articles describing health research evidence compared to the number of articles describing policy priorities and policy dialogues. Yet only 31 of the articles describing health research evidence mentioned systematic reviews, which are considered a logical unit to be the focus of efforts to support research use in health systems,[19] and only 27 articles described research regarding health systems[20]. Also, few articles described policy dialogues involving policymakers, researchers, and stakeholders, which are an important mechanism to support interactions among these groups and thereby encourage research use[5]. While a large number of articles were identified for certain countries like China and Uganda, many other jurisdictions appeared to have little print media coverage related to the three topics, and particularly with regards to policy priorities and policy dialogues. We found three or fewer articles about these topics in 30 and 32 of the jurisdictions, respectively.

### Strengths and limitations

Our study has three main strengths: 1) we used LexisNexis Academic, the largest and most comprehensive database for published news articles; 2) we developed search algorithms that can be replicated in future analyses; and 3) we reviewed the full text of all 32,938 articles to determine their eligibility for inclusion, as opposed to determining eligibility based only on examining article titles. Our study has four main limitations: 1) *Major World Publications *only includes English-language articles, which means that jurisdictions in some regions (e.g., Latin America) are covered through services like BBC Monitoring Latin America, rather than through local news sources; 2) we did not analyze regional and local news sources that were not included in LexisNexis, which may be an important source of media coverage for some jurisdictions (however, our pilot work had identified substantial challenges in using these sources, including the frequent lack of a searchable interface on local publication databases); 3) we only consulted print media, whereas television and radio may play a large role in reporting on health issues in some contexts (e.g., radio in Africa); and 4) the study was largely conducted by a single rater (i.e., one rater led the analysis for Africa, the Americas, and Asia, and another led the analysis for the Eastern Mediterranean), however, secondary reviewers were only involved in the analyses of Lebanon and Malaysia. Some of these weaknesses, however, can be addressed by focusing on changes over time. As part of the monitoring and evaluation of KT platforms in these 44 countries (or 47 jurisdictions), follow-up analyses will be conducted for 2009 and 2011. Future analyses will include several changes, such as adding the term "forum" in our search for policy dialogues (especially important for French-speaking countries) and coding for health systems research as an explicit part of the coding framework (rather than as an emergent thematic focus).

### Findings in relation to other studies

We are aware of three studies of print media coverage related to policy priorities, health research evidence, and policy dialogues regarding health issues, all of which were precursors to the study described here. The first study assessed the coverage potential of LexisNexis Academic and alternative regional and local print media sources for many of the jurisdictions we studied[15]. The second and third studies -- a media analysis of Cameroon and a media analysis of Asian jurisdictions - were also carried out by a single person using LexisNexis Academic, however, a second reviewer was not used for select jurisdictions and hence inter-rater reliability could not be assessed [18,21]. Based on experiences from the second study, we clarified our inclusion/exclusion criteria and made some modifications to our search strategy and coding protocol. Based on experiences from the third study, we chose to review the full texts of all articles initially returned by our search strategy, instead of assessing inclusion/exclusion based on the article titles.

### Implications for policy and research

Our study provides a window onto public debates regarding health issues, and our search strategy can be used in the future to profile how such discussions change over time. There remains, however, concern regarding the gaps in media coverage of *Major World Publications *in LexisNexis Academic. These issues may be addressed by incorporating analyses of local newspapers and non-English sources, though this may not be feasible (e.g., because many newspaper websites do not contain searchable databases) or methodologically appropriate (e.g., because what can be searched or accessed may not allow for meaningful cross-country comparisons). Considering the many roles played by the news media, KT platforms and others interested in supporting evidence-informed health systems need to recognize the importance of engaging the media in pursuing their desired outcomes. News media need to recognize the importance of their role in supporting evidence-informed health systems.

## Conclusions

In many countries the print media, at least as captured in a global database, are largely silent about three topics central to evidence-informed health systems. These findings suggest the need for proactive-media engagement strategies. Such strategies may include KT platforms hiring journalists if sufficient resources are available, capacity building in media relations for KT platform staff and utilizing social networking tools.

## Competing interests

The authors declare that they have no competing interests.

## Authors' contributions

AC developed the study methodology, screened articles (from Africa, the Americas, and Asia) for inclusion, coded articles (from Africa, the Americas, Asia, and the Eastern Mediterranean), and drafted the paper. JNL developed the study concept and objectives, and contributed to writing the draft. AH screened articles (from Africa and the Eastern Mediterranean) for inclusion, and coded articles (from the Eastern Mediterranean). FE-J screened articles (from the Eastern Mediterranean) for inclusion. JS screened articles (from Malaysia) for inclusion, and coded articles (Malaysia). NS and the KTPET provided input during study execution based on the respective regional expertise. All authors revised the paper before submission.

## Supplementary Material

Additional file 1 **LexisNexis Academic search algorithms**. Click here for file

Additional file 2 **Search algorithm returns and the number of articles included**. Click here for file
