# Supplementary material for: Climate for evidence-informed health systems: A print media analysis in 44 low- and middle-income countries that host knowledge-translation platforms
Source: Health Res Policy Syst. 2011 Feb 8;9:7. doi: 10.1186/1478-4505-9-7 (PMC3045990; doi:10.1186/1478-4505-9-7)
Supplement: Additional file 1 — LexisNexis Academic search algorithms. [file 1478-4505-9-7-S1.DOC]

Additional file 1 – LexisNexis Academic search algorithms

| **Category** | **Search phase** | **Algorithm** |
| --- | --- | --- |
| **Common search features** | All | 1. Under *Search* tab, select *News* 2. Under *Select Source*, select *Major World Publications* 3. Under *Specify Date*, input *Date is between*, *Jan 1 2007* and *Dec 31 2007* |
| **Policy priorities** | 1 | Input *Health* (anywhere in document) AND [*Jurisdiction*] (in indexing – any reference) AND *Priori!* (anywhere in document) |
|  | 2 | Same details as phase one, except input *Health* (in indexing – any reference) as opposed to *Health* (anywhere in document) |
|  | 3 | Same details as phase two, except input *Priori!* (in headline & lead paragraphs), as opposed to *Priori*! (anywhere in document) |
| **Health research evidence** | 1 | Input *Health* (anywhere in document) AND [*Jurisdiction*] (in indexing – any reference) AND (*Research* OR *Science* OR *Evidence*) (in indexing – any reference) OR (*Stud!* OR *Review* OR *Synthe!*)(anywhere in document) |
|  | 2 | Same details as phase one, except input *Health* (in indexing – any reference) as opposed to *Health* (anywhere in document) |
|  | 3 | Same details as phase two, except search for [*Research* OR *Science* OR *Evidence*] (in indexing – any reference) AND [*Stud!* OR *Review* OR *Synthe!*] (anywhere in document), instead of using OR connector |
| **Policy dialogues** | 1 | Input *Health* (in indexing – any reference) AND [*Jurisdiction*] (in indexing – any reference) AND *Meet!* OR *Conference* OR *Workshop* (anywhere in document) |
|  | 2 | Same details as phase one, except input *Conference* (in indexing – any reference) as opposed to *Conference* (anywhere in document) |
|  | 3 | Same details as phase two, except input *Meet*! (in indexing – any reference) as opposed to *Meet*! (anywhere in document) |
