# Supplementary material for: Climate for evidence-informed health systems: A print media analysis in 44 low- and middle-income countries that host knowledge-translation platforms
Source: Health Res Policy Syst. 2011 Feb 8;9:7. doi: 10.1186/1478-4505-9-7 (PMC3045990; doi:10.1186/1478-4505-9-7)
Supplement: Additional file 2 — Search algorithm returns and the number of articles included. [file 1478-4505-9-7-S2.DOC]

***Additional file 2 –*** Search algorithm returns and the number of articles included

| KT platform countries (or jurisdictions) | **Policy priorities** | |  | **Health research evidence** | | | |  | **Policy dialogues** | | | |
| --- | --- | --- | --- | --- | --- | --- | --- | --- | --- | --- | --- | --- |
|  | **Phase 1 returns** | **Articles included** |  | **Phase 1 returns** | **Phase 2 returns** | **Phase 3 returns** | **Articles included** |  | **Phase 1 returns** | **Phase 2 returns** | **Phase 3 returns** | **Articles included** |
| Burkina Faso | 35 | 1 |  | 117 | - | - | 13 |  | 48 | - | - | 2 |
| Burundi | 37 | 0 |  | 101 | - | - | 5 |  | 51 | - | - | 5 |
| Cameroon | 82 | 10 |  | 218 | - | - | 15 |  | 75 | - | - | 8 |
| Central African Republic | 7 | 0 |  | 9 | - | - | 0 |  | 10 | - | - | 0 |
| Ethiopia | 129 | 12 |  | 480 | - | - | 14 |  | 219 | - | - | 12 |
| Kenya | 369 | 16 |  | * | 750 | - | 62 |  | 703 | - | - | 17 |
| Mali | 26 | 1 |  | 101 | - | - | 15 |  | 43 | - | - | 0 |
| Mozambique | 101 | 9 |  | 297 | - | - | 22 |  | 144 | - | - | 6 |
| Nigeria‡ | 691 | 26 |  | * | 680 | - | 45 |  | 811 | - | - | 21 |
| Rwanda | 145 | 19 |  | 511 | - | - | 23 |  | 244 | - | - | 27 |
| Tanzania | 104 | 7 |  | 528 | - | - | 36 |  | 203 | - | - | 2 |
| Uganda | 366 | 19 |  | * | 806 | - | 105 |  | 774 | - | - | 50 |
| Zambia | 104 | 5 |  | 352 | - | - | 34 |  | 160 | - | - | 4 |
| **All KT platforms in Africa** | **2196** | **125** |  | **2714** | **2236** | **0** | **389** |  | **3485** | **0** | **0** | **154** |
| Argentina | 32 | 0 |  | 296 | - | - | 31 |  | 45 | - | - | 0 |
| Bolivia | 11 | 0 |  | 50 | - | - | 2 |  | 9 | - | - | 0 |
| Brazil | 124 | 3 |  | 1035 | - | - | 205 |  | 178 | - | - | 2 |
| Chile | 20 | 1 |  | 158 | - | - | 19 |  | 28 | - | - | 0 |
| Colombia | 28 | 0 |  | 102 | - | - | 9 |  | 27 | - | - | 1 |
| Costa Rica | 10 | 0 |  | 78 | - | - | 9 |  | 29 | - | - | 1 |
| Mexico§ | 117 | 1 |  | 875 | - | - | 67 |  | 202 | - | - | 2 |
| Paraguay | 6 | 0 |  | 22 | - | - | 0 |  | 7 | - | - | 0 |
| Puerto Rico | 6 | 0 |  | 115 | - | - | 12 |  | 31 | - | - | 0 |
| Trinidad and Tobago | 9 | 1 |  | 43 | - | - | 2 |  | 14 | - | - | 2 |
| **All KT platforms in the Americas** | **363** | **6** |  | **2774** | **0** | **0** | **356** |  | **570** | **0** | **0** | **8** |
| Bangladesh‡ | 37 | 0 |  | 263 | - | - | 43 |  | 63 | - | - | 0 |
| China | 1017 | 46 |  | * | † | 991 | 294 |  | * | † | 1025 | 48 |
| Beijing | 337 | 2 |  | * | 732 | - | 26 |  | 871 | - | - | 0 |
| Shandong | 19 | 0 |  | 125 | - | - | 15 |  | 52 | - | - | 0 |
| Sichuan | 13 | 0 |  | 93 | - | - | 12 |  | 30 | - | - | 0 |
| Kyrgyzstan‡ | 11 | 1 |  | 27 | - | - | 0 |  | 20 | - | - | 0 |
| Laos PDR | 14 | 0 |  | 81 | - | - | 2 |  | 25 | - | - | 0 |
| Malaysia | 181 | 13 |  | * | 683 | - | 64 |  | 476 | - | - | 29 |
| Philippines | 217 | 22 |  | 976 | - | - | 36 |  | 313 | - | - | 16 |
| Vietnam | 82 | 5 |  | 611 | - | - | 31 |  | 133 | - | - | 11 |
| **All KT platforms in Asia** | **1928** | **89** |  | **2176** | **1415** | **991** | **523** |  | **1983** | **0** | **1025** | **104** |
| Bahrain | 4 | 1 |  | 38 | - | - | 1 |  | 8 | - | - | 0 |
| Egypt | 124 | 6 |  | 661 | - | - | 33 |  | 143 | - | - | 2 |
| Iran | 174 | 13 |  | 1089 | - | - | 74 |  | 207 | - | - | 4 |
| Iraq | 942 | 0 |  | * | 1127 | - | 12 |  | 870 | - | - | 3 |
| Jordan | 50 | 3 |  | 248 | - | - | 5 |  | 70 | - | - | 2 |
| Lebanon | 45 | 1 |  | 319 | - | - | 20 |  | 76 | - | - | 1 |
| Libya | 27 | 0 |  | 135 | - | - | 8 |  | 41 | - | - | 1 |
| Morocco | 22 | 0 |  | 156 | - | - | 7 |  | 29 | - | - | 0 |
| Oman | 4 | 0 |  | 40 | - | - | 5 |  | 4 | - | - | 0 |
| Pakistan | 135 | 9 |  | 814 | - | - | 23 |  | 246 | - | - | 3 |
| Sudan | 133 | 9 |  | 480 | - | - | 7 |  | 167 | - | - | 6 |
| Syria | 48 | 0 |  | 255 | - | - | 3 |  | 67 | - | - | 1 |
| Tunisia | 4 | 2 |  | 30 | - | - | 2 |  | 9 | - | - | 1 |
| Yemen | 3 | 0 |  | 36 | - | - | 0 |  | 2 | - | - | 0 |
| **All KT platforms in the Eastern Mediterranean** | **1715** | **44** |  | **4301** | **1127** | **0** | **200** |  | **1939** | **0** | **0** | **24** |

*Phase 1 search returned more than 1500 articles

†Phase 2 search returned more than 1500 articles

‡KT platforms in these countries are not part of EVIPNet but are undertaking very similar activities and producing similar outputs

§Mexico has two WHO-sponsored EVIPNets (one at the national level, and one focused on the US/Mexico border region) and another KT platform sponsored by a domestic institution (Mexico’s National Institute of Public Health); the US/Mexico border region was not included in this study as it would have required an analysis for all of the US, in addition to Mexico.
